# Severe Primary Varicella-Zoster Virus Meningitis With Dual Cranial Neuropathy (Cranial Nerve VII and Cranial Nerve XII) in a 67-Year-Old Man With Post-traumatic Asplenia: A Case Report

**DOI:** 10.7759/cureus.117649

**Published:** 2026-10-08

**Authors:** Khatia Ostatishvili, Tamari Chaprava, Sopio Babutsidze, Paata Nadimashvili, Alexandre Goginava

**Affiliations:** 1 Microbiology, Tbilisi State Medical University, Tbilisi, GEO; 2 Infectious Diseases, Global Med University Clinic, Tbilisi, GEO; 3 Infectious Diseases, Pineo Medical Ecosystem, Tbilisi, GEO; 4 Healthcare Management, Global Med University Clinic, Tbilisi, GEO; 5 Neurology, Global Med University Clinic, Tbilisi, GEO; 6 Infectious Diseases, German Hospital, Tbilisi, GEO

**Keywords:** acyclovir, cranial neuropathy, facial nerve palsy, hypoglossal nerve palsy, neuroinvasive varicella-zoster virus, valacyclovir, varicella-zoster virus, varicella-zoster virus meningitis

## Abstract

Primary varicella-zoster virus (VZV) infection in adults can cause severe systemic and neuroinvasive complications, particularly in older patients and those with impaired splenic function. A 67‑year‑old man with post-traumatic asplenia and hypertension presented on day five of varicella with acute viral meningitis, right peripheral facial nerve palsy (cranial nerve (CN) VII), and right hypoglossal nerve palsy (CN XII). Cerebrospinal fluid (CSF) analysis, including multiplex polymerase chain reaction (PCR) testing, confirmed VZV infection with lymphocytic pleocytosis and elevated protein levels. Contrast-enhanced magnetic resonance imaging of the brain revealed localized leptomeningeal enhancement without brainstem or parenchymal involvement. Following diagnosis, an appropriate course of intravenous and oral antiviral therapy was administered, resulting in a marked clinical and laboratory response. Oral pregabalin was initiated and titrated for persistent subacute neuropathic pain. Meningitis and systemic infection resolved, and the patient was discharged in stable condition with outpatient follow‑up for CN recovery and neuralgia management. This case highlights the risk of severe neuroinvasive VZV in older asplenic patients and supports early CSF PCR testing and prompt initiation of extended antiviral therapy in high‑risk individuals.

## Introduction

Primary varicella‑zoster virus (VZV) infection in immunocompetent children usually presents as varicella (chickenpox), a self‑limiting exanthematous disease [1]. However, when primary infection occurs in adults, particularly in individuals with altered immune function, such as those with post‑traumatic asplenia or immunosenescence, it carries a significantly higher risk of severe systemic and neuroinvasive manifestations [1,2]. VZV exhibits marked neurotropism, establishing latency within sensory ganglia following primary infection and retaining the potential to disseminate into the central nervous system (CNS) [2,3].

While primary VZV infection typically presents as varicella, acute neuroinvasive complications in adults, manifesting concurrently as viral meningitis, peripheral facial nerve palsy (cranial nerve (CN) VII), and hypoglossal nerve palsy (CN XII), remain extraordinarily rare [4].

Post-traumatic asplenia, much like functional or anatomical asplenia of any etiology, impairs immune clearance and early viral control mechanisms, largely due to the loss of the spleen's critical immunological role in rapid immunoglobulin M production and opsonizing antibody synthesis [5]. Furthermore, delayed presentation and the resulting lag in initiating targeted antiviral therapy can exacerbate acute neuroinflammation, predisposing patients to multifocal cranial neuritis and prolonged subacute neuropathic pain [6].

Here, we present a rare clinical case of severe primary VZV meningitis complicated by dual cranial neuropathies (CN VII and CN XII) and subacute neuralgia in a 67‑year‑old man with post‑traumatic asplenia. This report highlights the clinical impact of delayed antiviral intervention, the diagnostic utility of cerebrospinal fluid (CSF) polymerase chain reaction (PCR), and the necessity of targeted multidisciplinary management in high‑risk patients.

## Case presentation

A 67‑year‑old male presented to our emergency department on day five of illness with severe headache, progressive facial asymmetry, speech difficulty, and a widespread cutaneous eruption. His medical history was notable for secondary post‑traumatic asplenia resulting from a prior splenectomy and long‑standing essential hypertension managed with oral antihypertensive therapy. Notably, the patient had no history of varicella‑zoster vaccination and reported no documented or recalled history of childhood varicella (chickenpox) infection.

Five days before admission, the patient experienced a sudden, severe headache, which he initially attributed to cold air exposure. An outpatient neurological consultation resulted in a prescription for non-steroidal anti-inflammatory drugs. On day two of illness, a polymorphic vesicular rash appeared on his neck, subsequently spreading to the scalp, face, chest, and upper extremities. By days three to four, the patient developed acute facial asymmetry and mild dysarthria, prompting emergency medical services activation and transfer to our tertiary facility.

Upon admission on day five, the patient presented in acute distress with a body temperature of 38.3°C, a heart rate of 105 beats/minute, a blood pressure of 140/75 mmHg, and an oxygen saturation of 95% on ambient air. Pulmonary auscultation revealed bilateral vesicular breath sounds accompanied by scattered dry crackles. Dermatological examination unveiled a profuse, polymorphic vesicular eruption distributed predominantly across the neck, scalp, face, anterior chest, and upper extremities (Figure 1).

**Figure 1:**
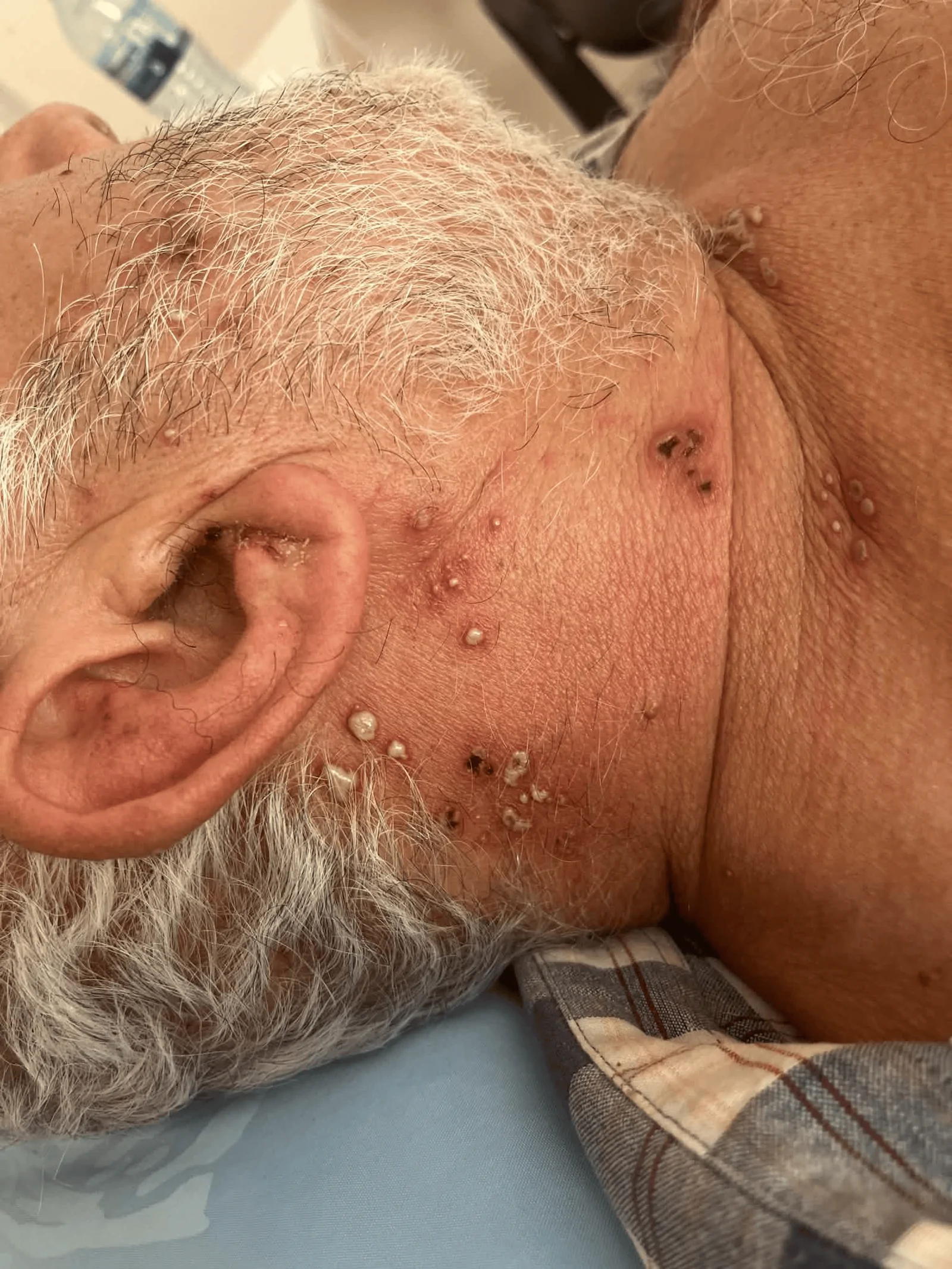
Polymorphic vesicular and crusted eruption involving the right preauricular area, scalp, neck, and anterior chest. Widespread polymorphic vesicles and crusting over the right hemiface, scalp, neck, and anterior chest.

Neurologically, the patient appeared obtunded with delayed responsiveness. Pronounced nuchal rigidity was observed alongside strongly positive Kernig’s signs bilaterally. CN evaluation revealed striking deficits, including a right-sided peripheral facial nerve palsy (CN VII) characterized by lagophthalmos and sluggish corneal reflexes, coupled with soft palate weakness. Furthermore, tongue protrusion elicited a distinct rightward deviation, pointing to a concurrent right hypoglossal nerve palsy (CN XII) (Figure 2). Deep tendon reflexes remained brisk and symmetrical, while Babinski’s sign was positive bilaterally.

**Figure 2:**
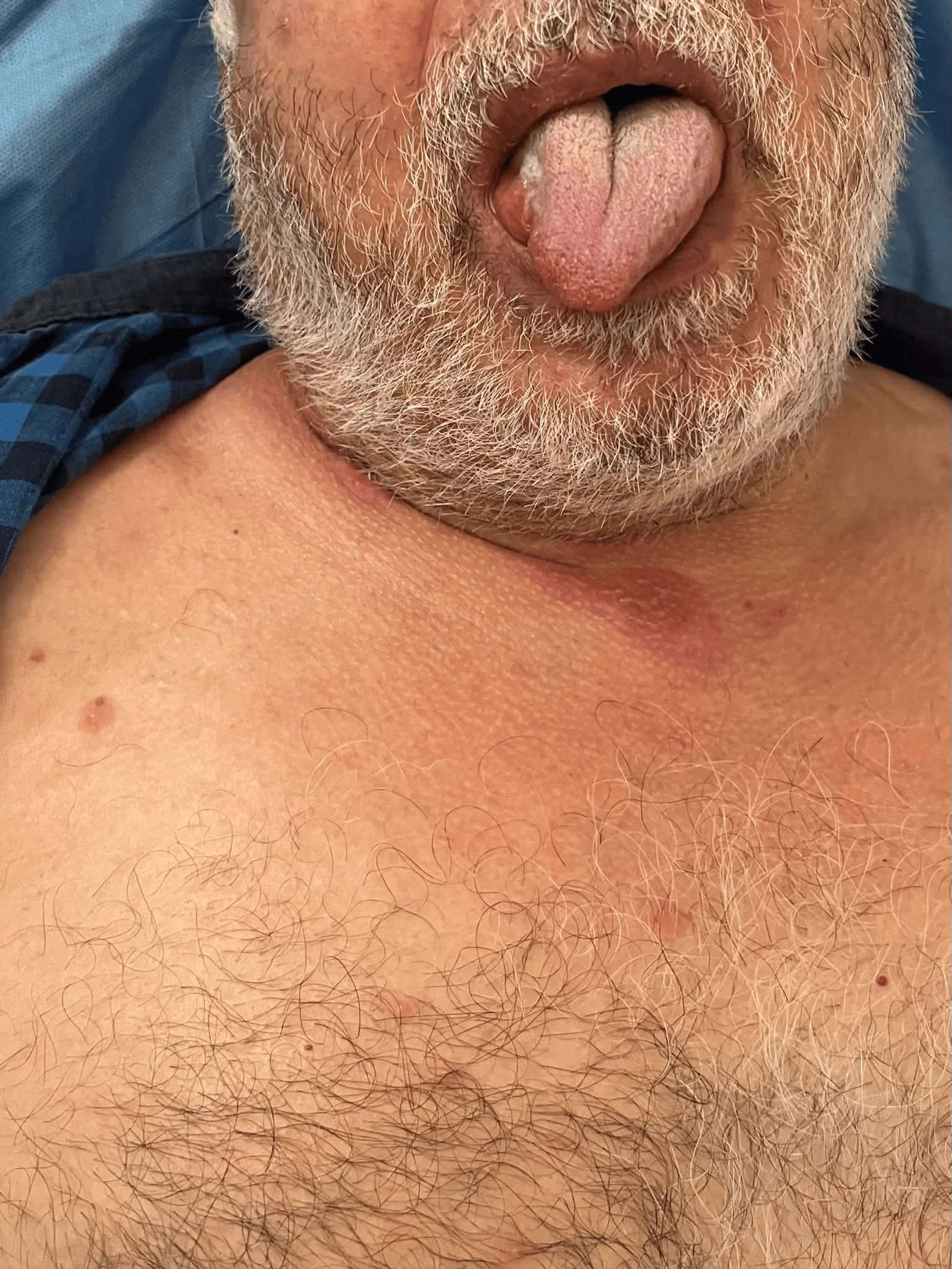
Clinical photograph demonstrating rightward tongue deviation indicating hypoglossal nerve (cranial nerve XII) involvement. Tongue protrusion showing distinct rightward deviation.

Initial laboratory evaluation demonstrated a white blood cell count of 7.41 × 10^9^/L with 64.7% neutrophils and 27.5% lymphocytes. C‑reactive protein was elevated at 23.74 mg/L (subsequently rising to 33.30 mg/L on day eight). Serum ferritin was 340.9 ng/mL and lactate dehydrogenase was 351.5 U/L. Renal function testing revealed mild serum creatinine elevation (102.9 µmol/L on admission, peaking at 156.0 µmol/L on day 11, before normalizing). Serological screening for hepatitis B surface antigen, anti‑hepatitis C virus, anti‑human immunodeficiency virus 1+2, and syphilis was negative.

Non‑contrast computed tomography (CT) of the head ruled out acute intracranial hemorrhages or space‑occupying lesions. Magnetic resonance imaging (MRI) of the brain without contrast was unremarkable for acute parenchymal lesions or ischemic stroke (revealing only a small non-specific gliotic focus in the right frontal lobe). However, following intravenous contrast administration (post-contrast T1-weighted/fluid-attenuated inversion recovery images), localized leptomeningeal enhancement was clearly demonstrated within the left frontoparietal sulci, consistent with focal inflammatory meningeal reaction (Figure 3). In addition, a hyperintense signal in the right mastoid air cells was noted, indicating right-sided mastoiditis.

**Figure 3:**
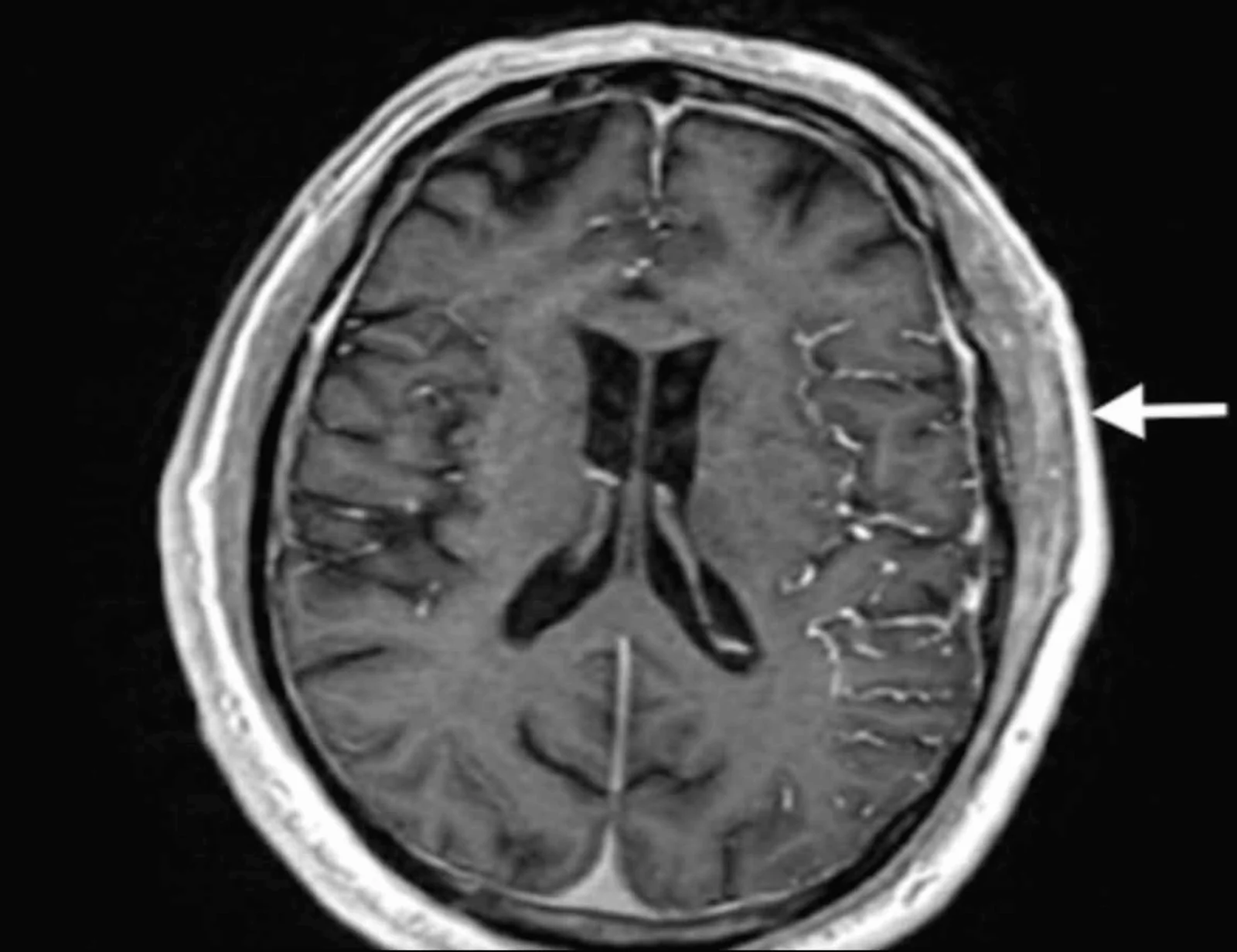
Magnetic resonance imaging (MRI) of the brain demonstrating localized leptomeningeal enhancement. Axial post-contrast T1-weighted/fluid-attenuated inversion recovery MRI of the brain showing localized leptomeningeal enhancement within the left frontoparietal sulci (indicated by the white arrow), consistent with focal inflammatory meningeal reaction.

Lumbar puncture performed on admission yielded clear CSF. CSF analysis revealed moderate lymphocytic/mononuclear pleocytosis with 267 cells/µL (predominantly lymphocytes and monocytes) and an elevated protein level of 1.74 g/L, with normal glucose concentration. CSF multiplex PCR testing was negative for herpes simplex virus types 1 and 2 (HSV‑1, HSV‑2) but positive for VZV DNA, establishing the definitive diagnosis of acute VZV meningitis.

Targeted high‑dose antiviral therapy was immediately initiated on admission (day five of illness) with intravenous acyclovir (10 mg/kg three times daily, maximum dose of 1,000 mg) for two weeks. Concurrently, short‑term adjunctive corticosteroid therapy was administered alongside the antiviral agent to mitigate severe neuroinflammatory and meningeal edema. Blood pressure was closely monitored and continuously regulated using his baseline oral antihypertensive regimen to manage long-standing essential hypertension. Furthermore, the patient’s medical history of secondary post‑traumatic asplenia was carefully factored into his comprehensive care plan. Clinical improvement became evident progressively during therapy: headache intensity markedly decreased by days seven to eight, meningeal signs gradually resolved by day 10, and cutaneous lesions fully crusted over by day 19, alongside partial recovery of facial asymmetry. To ensure sustained virologic suppression, parenteral therapy was followed by oral valacyclovir (1,000 mg three times daily) for an additional two weeks, completing a total four-week antiviral course. Follow‑up CSF analysis on treatment day 10 showed a 2.5‑fold reduction in pleocytosis (100 cells/µL) with persistent mild protein elevation. A final control CSF puncture on day 14 demonstrated marked inflammatory clearance, with pleocytosis decreasing to 52 cells/µL and a significant drop in CSF protein, confirming resolution of the acute inflammatory phase (Table 1).

**Table 1:** CSF and selected serum parameters during hospitalization. Treatment Day 10 and control/clearance days correspond to the dates of follow-up lumbar punctures. Discharge values reflect clinical status at hospital discharge. “Moderately elevated” and “Marked clearance” describe relative CSF protein changes compared with admission values (numeric protein values are available in laboratory records). Reference ranges are provided for comparison; “—” indicates the parameter was not measured or not applicable at that time point. Admission = hospital presentation (day five of illness). CSF = cerebrospinal fluid; WBC = white blood cells; VZV = varicella-zoster virus; PCR = polymerase chain reaction; CRP = C-reactive protein

Parameter	Admission (day 5)	Treatment day 10 (July 20)	Control/Clearance (day 14, July 24)	Discharge (day 19, July 29)	Reference range
CSF WBC (pleocytosis)	267 cells/µL	100 cells/µL	52 cells/µL	—	<5 cells/µL
CSF protein	1.74 g/L	1.15 g/L	0.65 g/L	—	0.15–0.45 g/L
CSF glucose	Normal	Normal	Normal	—	2.2–3.9 mmol/L
CSF VZV PCR	Positive	Not repeated	Not repeated	—	Negative
Serum CRP	23.74 mg/L	33.30 mg/L (Day 8)	Normalizing	Normal	<5.0 mg/L
Serum creatinine	102.9 µmol/L	156.0 µmol/L (Day 11)	79.24 µmol/L	107.7 µmol/L	62–115 µmol/L

Prolonged inpatient management was strictly required due to the patient’s high-risk status with post-traumatic asplenia, the severe presentation of neuroinvasive disease involving dual cranial neuropathies, and the critical clinical necessity to complete the full intravenous antiviral course and monitor CSF clearance through serial punctures before transitioning to outpatient oral therapy. By day 19 of hospitalization, cutaneous lesions had fully crusted over, and meningeal signs were completely absent. Systemic parameters stabilized. Neurologically, residual right facial asymmetry (CN VII) persisted, alongside rightward tongue deviation (CN XII) and localized right‑sided facial neuropathic pain (subacute neuralgia). Oral pregabalin was introduced and titrated for neuralgia control. The patient was discharged home in stable condition with outpatient follow‑up recommendations for CN recovery and pain management, displaying significant clinical and dermatological improvement upon discharge (Figure 4).

**Figure 4:**
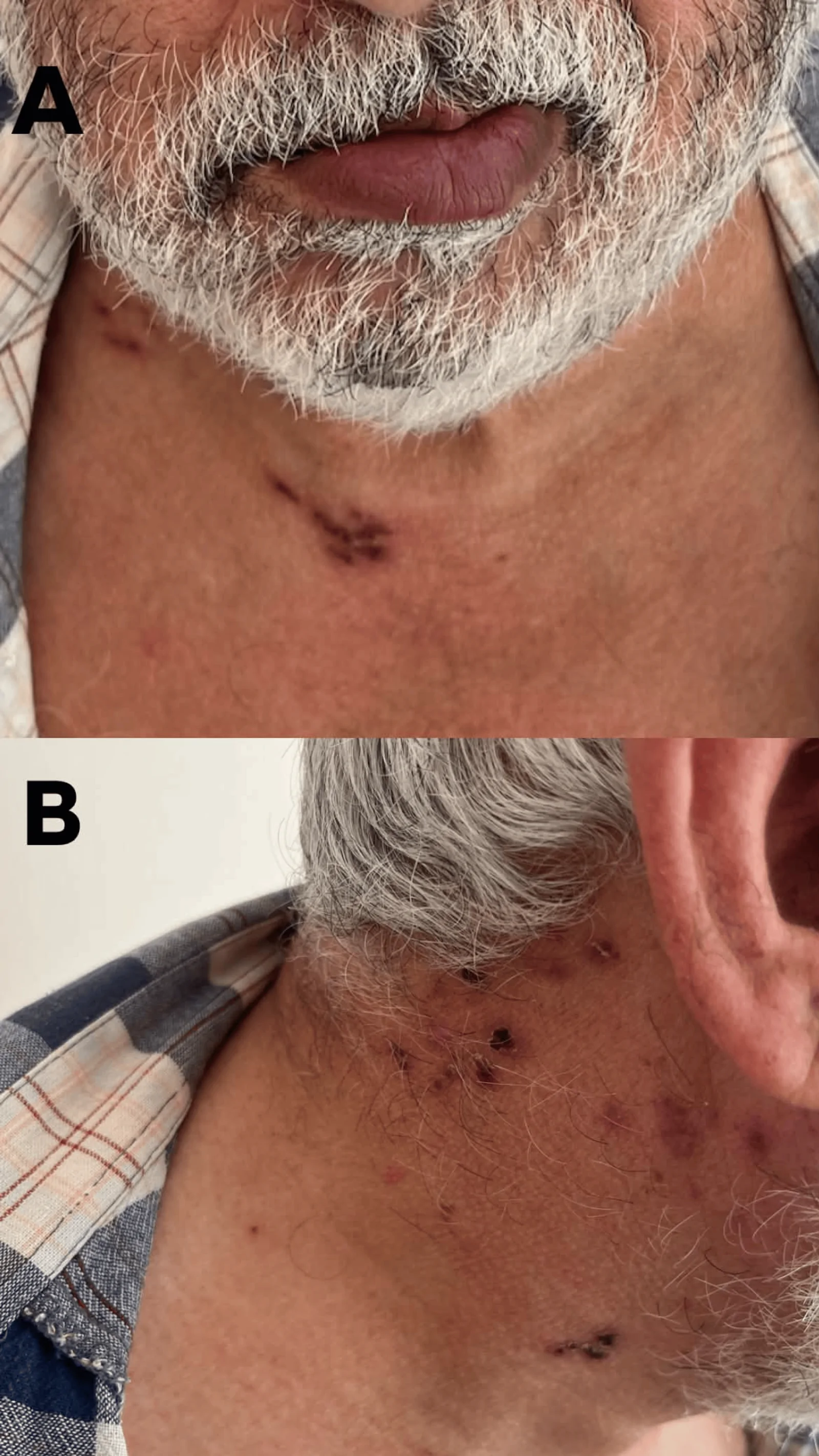
Clinical photographs at discharge demonstrating dermatological resolution and persistent neurological deficits. Clinical appearance on day 19 of hospitalization. (A) Frontal view showing minimal residual right facial asymmetry. (B) Lateral view of the neck depicting complete crusting and healing of the vesicular eruptions.

## Discussion

VZV is a neurotropic alpha‑herpesvirus that causes primary varicella (chickenpox) and subsequently establishes lifelong latency within the CN, dorsal root, and autonomic ganglia [1,3]. While primary VZV infection in adults typically presents with an exanthematous rash, severe neuroinvasion can occur, resulting in acute complications including aseptic meningitis, encephalitis, vasculopathy, myelitis, and polyneuritis cranialis [1,2].

The presentation of primary VZV infection involving a simultaneous widespread varicella eruption, acute aseptic meningitis, and multiple cranial neuropathies, specifically involving the facial nerve (CN VII) and hypoglossal nerve (CN XII), is exceptionally rare in older adults without overt systemic immunosuppression [2,4]. In our patient, the diagnosis was definitively established by multiplex PCR of the CSF, which identified VZV DNA despite unremarkable initial systemic inflammatory markers. This underscores the indispensable role of early molecular CSF analysis in patients presenting with acute meningeal symptoms and CN deficits, even when routine blood tests are non‑diagnostic.

A notable feature of this case is the patient’s unconfirmed history of childhood varicella and complete absence of varicella‑zoster vaccination. Adults lacking vaccine‑induced immunity who experience primary VZV infection, or an atypical presentation following subclinical childhood exposure, are at an increased risk for severe neuroinvasive disease [1,4]. This risk was compounded by the patient’s history of post‑traumatic splenectomy. Although asplenia predominantly increases susceptibility to encapsulated bacterial infections, it also perturbs immune homeostasis and may impair T‑cell-mediated surveillance, predisposing patients to severe viral neuroinvasion and multi‑nerve dissemination [5].

The combined involvement of the hypoglossal nerve (CN XII) and peripheral facial nerve (CN VII) represents a distinct neurological manifestation. Hypoglossal palsy secondary to VZV is exceedingly uncommon [7]. The pathogenesis of multiple CN involvement in VZV meningitis is likely multifactorial, including direct transaxonal viral spread, retrograde transport, and localized inflammatory neuritis secondary to VZV vasculitis or subarachnoid inflammatory exudates [3]. Brain MRI in this case showed localized leptomeningeal enhancement of the left frontal and parietal sulci, consistent with focal inflammatory meningeal reaction. Although the brainstem was structurally intact on MRI and leptomeningeal enhancement was predominantly visible in the left frontoparietal region, the patient presented with right-sided peripheral cranial neuropathies. In the context of primary VZV infection, neurological manifestations are driven by diffuse, multifocal meningeal involvement and viremia-mediated dissemination rather than isolated focal parenchymal lesions. While standard neuroimaging may not always resolve subtle or microscopic basal meningeal inflammation due to technical limitations or bone artifacts, VZV-induced inflammatory processes frequently extend across the subarachnoid spaces and skull base cisterns. Consequently, CNs traversing these compartments are vulnerable to localized inflammatory exudates and secondary microvascular injury (vasa nervorum vasculitis), leading to cranial neuropathies that manifest independently of direct, macroscopic brainstem parenchymal lesions on standard MRI sequences.

Current evidence‑based guidance supports high‑dose intravenous acyclovir (10 mg/kg every eight hours) as the mainstay of therapy for VZV CNS infections, targeting viral DNA polymerase, mitigating vascular injury, and reducing long‑term neurological sequelae [6]. While standard guidelines recommend 10 to 14 days of parenteral therapy in immunocompetent hosts, optimal dosing and duration in high-risk asplenic patients often warrant extended, tailored regimens. Transitioning from two weeks of parenteral high-dose acyclovir to two additional weeks of oral valacyclovir ensures sustained virologic suppression and prevents disease relapse. Prompt antiviral initiation in this patient correlated with clinical stabilization and serial CSF improvement: pleocytosis fell to 100 cells/µL by treatment day 10 and to 52 cells/µL by day 14, paralleling cutaneous lesion crusting and clinical recovery.

Short‑term adjunctive corticosteroid therapy was used to reduce perineural edema and meningeal inflammation [7]. Despite virologic and inflammatory resolution, residual right facial weakness (CN VII), tongue deviation (CN XII), and subacute neuropathic pain persisted at discharge; these were managed with oral pregabalin [8]. Long‑term outpatient follow‑up, pain management, and neurorehabilitation are essential to optimize CN recovery.

## Conclusions

VZV neuroinvasion can present as a severe multisystem disorder characterized by widespread varicella, aseptic meningitis, and cranial polyneuropathy involving the facial nerve (CN VII) and hypoglossal nerve (CN XII). Clinicians should maintain a high index of suspicion for neuroinvasive VZV infection in adults presenting with unexplained headache and acute cranial deficits, particularly in those with functional or anatomical immunodeficiency such as asplenia. Early CSF multiplex PCR testing is crucial for prompt diagnosis. Immediate initiation of high‑dose intravenous acyclovir followed by tailored oral antiviral step-down therapy, alongside serial CSF monitoring, is essential to achieve clinical improvement, prevent disease relapse, and optimize clinical outcomes in high-risk patients.
